# Preparation and Properties of Flexible Phenolic Silicone Hybrid Aerogels for Thermal Insulation

**DOI:** 10.3390/molecules29204942

**Published:** 2024-10-18

**Authors:** Danni Ye, Hongli Lv, Zhenrong Zheng, Lijuan Luo

**Affiliations:** 1 School of Textile Science and Engineering, Tiangong University, Tianjin 300387, China; yedannitjpu@163.com; 2 SWOTO (Beijing) Protection Technology Co., Ltd., Beijing 100041, China; wyyx22211@163.com; 3 Aerospace Research Institute of Materials and Processing Technology, Beijing 100076, China; luolijuan_1010@163.com

**Keywords:** silicon-based aerogel, phenolic aerogel, isocyanate coupling agent

## Abstract

In order to prepare flexible thermal protection aerogel materials, using dimethyldimethoxysilane (DMDMS) and methyltrimethoxysilane (MTMS) as co-precursors, isocyanate-propyltrimethoxysilane (CFS-006) was added to the co-precursor as a coupling agent, and resorcinol and formaldehyde were added to the sol solution to prepare a phenolic silicone hybrid aerogel (FAS) by the sol–gel method. The prepared FAS aerogel had no phase separation problem, the density was only 0.118 g/cm^3^, the hydrophobic angle reached 155.3°, and it had certain flexibility. It could be compressed to 70% and still be restored to its original state. The FAS aerogel also had a low thermal conductivity of 0.0318 W/(m·K) and good high temperature insulation. The introduction of phenolic groups improved thermal stability; Tmax increased to 643.7 °C, and the residual carbon rate was 24.5%. This work has positive significance for the future combination of aerogels and textiles in the preparation of firefighting protective clothing.

## 1. Introduction

An aerogel is a nanoscale solid substance formed by using gas to replace the liquid-phase components inside the wet gel-network structure. It has the characteristics of ultra-low density, low thermal conductivity, and a nanoporous structure [1]. There are various kinds of aerogels. They includes inorganic aerogel, organic aerogel, carbon aerogel, multi-component aerogel, etc. [2,3]. Currently, silicon-based aerogel has been studied deeply and applied in a relatively mature way: it is widely used in thermal insulation, adsorption, electromagnetic shielding, photocatalysis, and other fields of technology [4,5].

**Figure 1:**
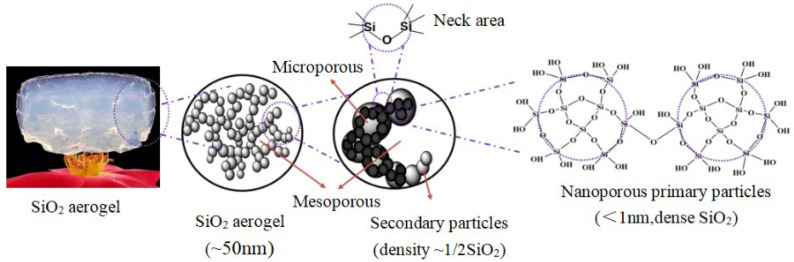
Appearance [12] and Microstructure of porous SiO_2_ aerogels.

The microstructure of silicon-based aerogel prepared by traditional methods is mainly composed of primary and secondary SiO_2_ particles, with a nacre-chain network composed of loosely packed and bonded particles or fibers [6] (Figure 1). The inherent weak strain of the silicon–oxygen covalent bond is one of the reasons for the poor flexibility of aerogels [7]. In order to reduce the dense silico–oxygen structure, multi-functional alkols can be selected as the silicon source precursors; commonly used compounds are methyl trimethoxy-silane (MTMS), dimethyl dimethoxy-silane (DMDMS), vinyl trimethoxy-silane (VTMS), etc. [8,9,10,11]. Such precursors can not only reduce the number of hydroxyl groups formed during hydrolysis but also generate a large number of silicon-carbon bonds, and the mutual repulsion between the organic groups can give the aerogel a certain flexibility.

**Figure 2:**
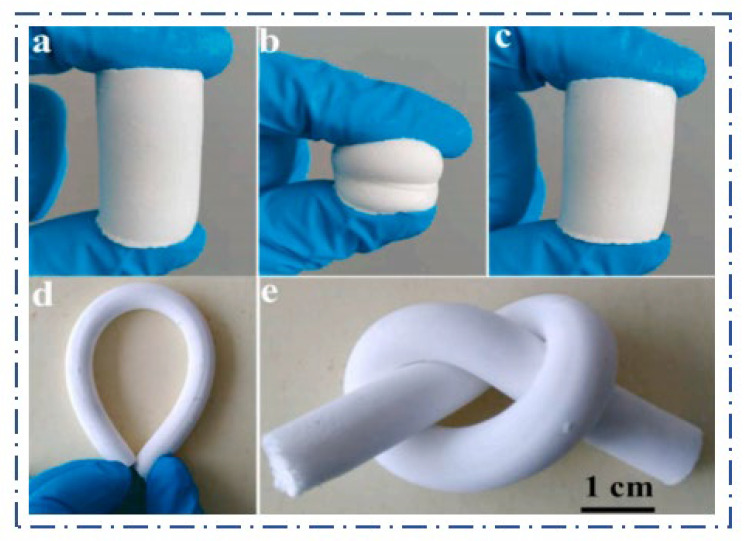
Ref. [17]. Flexibility of PMSA: (**a**–**c**) PMSA restored to shape after acupressure and (**d**,**e**) PMSA can be bent and twisted.

Liu et al. [13] prepared SiO_2_ aerogels with MTMS and DMDMS as co-precursors that had good mechanical strength, and high compressive strength of up to 40 kPa was obtained under 75% strain. Ma et al. [14] used MTMS and DMDMS as silica sources and used an amphiphilic silica monomer, triethoxysilane end-capped poly(ethylene oxide) (PEO-TES), as precursor and surfactant. The prepared aerogels can be restored to their original shape after twisting, curling, and folding, which demonstrated the flexibility of the aerogels. Li et al. [15] used VTMS and vinylmethyl dimethoxysilane (VMDMS) as silica source and silica sol was added as silica source system to prepare silica sol-reinforced flexible silica aerogels by ambient pressure drying. The maximum strain of the aerogels reached 80% in 10 compression–decompression cycles without destroying the structure of the aerogel, and the hydrophobic angle reached 145.7°. Li et al. [16] used methyl orthosilicate (TMOS) as silicon source and DMDMS as a doping term to prepare novel TMOS-DMDMS composite SiO_2_ aerogels. The compressive strength of the prepared aerogel was increased to 1.219 MPa. Guo et al. [17] synthesized highly flexible polymethylsilsesquioxane aerogel (PMSA) monoliths by using MTMS and DMDMS as co-precursors and ammonia as a base catalyst. The prepared aerogels had excellent compressibility and bending flexibility as shown in Figure 2.

The aerogel prepared by the method of using a co-precursor with the multifunctional silane with a methyl group as the silicon source overcomes the brittleness problem of the traditional silicone-based aerogel, the structure is stable, it does not fall off as a powder, and it has a certain degree of flexibility. However, it still has the problems of poor thermal stability and a low ablation residual rate in high-temperature environments, which cannot satisfy the increasing requirement of high temperature ablation resistance and other indexes of current heat insulation materials.

The carbonized layer formed by phenolic resin at high temperatures of 800~2500 °C has good heat resistance [18], which protects and insulates the internal materials. In addition, phenolic resin also has excellent high-temperature ablation resistance. Therefore, phenolic-based aerogels, as a typical ablation-resistant material, have good heat insulation, excellent mechanical properties, and high thermal stability and are widely used in the aerospace [19], construction [20], and thermal protection fields [21]. Despite the many advantages of phenolic resin aerogels, their low synthesis efficiency, high cost, complex synthesis process, and the large number of rigid benzene rings in phenolics make phenolic aerogels lose their flexibility, which still limits the application of phenolic aerogels [22]. Phenolic aerogels with both flexibility and ablation resistance have attracted much attention as a new type of multifunctional aerogel. There are no reactive groups between organic phenolic and inorganic SiO_2_ components, and phase separation will occur through physical network entangling. Chemical grafting can solve the problem of cross-linking difficulties.

In this paper, DMDMS and MTMS were used as co-precursors, CFS-006 was added to the co-precursor as a coupling agent, and resorcinol and formaldehyde were added to the sol solution to prepare a phenolic silicone hybrid aerogel (FAS) by the sol–gel method. Unhybridized aerogel prepared with the same co-precursor system was called DM aerogel. The microstructure, infrared spectroscopy, thermal insulation, and hydrophobic properties of the hybrid aerogels were characterized. The performance of FAS aerogel was compared with DM aerogel. This work has very important research value and can provide a new idea for the preparation of flexible and heat-insulating aerogel materials.

## 2. Results and Discussion

### 2.1. Reaction Mechanism of the DM Aerogel

**Figure 3:**
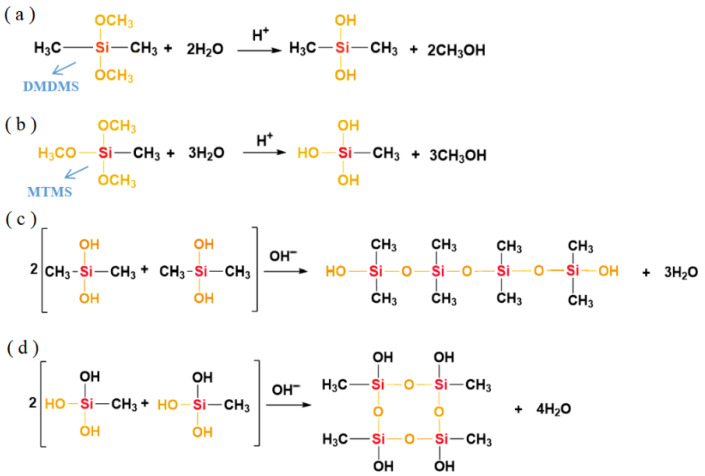
(**a**) Hydrolysis reaction of DMDMS; (**b**) hydrolysis reaction of MTMS; (**c**) polycondensation reaction of DMDMS; (**d**) polycondensation reaction of MTMS.

The hydrolysis polycondensation reaction mechanism of the DM aerogel is as follows [23]: DMDMS is a difunctional silane containing two groups that can participate in the reaction in each molecule, and MTMS is a trifunctional silane containing three reactive groups in each molecule. The hydrolysis reaction of DMDMS and MTMS occurs under an acidic catalyst (Figure 3a,b), where the methoxyl group is changed to a hydroxyl group, and the hydrolysis generates small molecule methanol.

**Figure 4:**
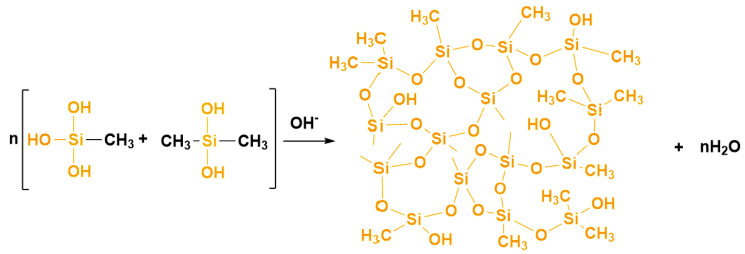
Polycondensation reaction of DMDMS and MTMS.

Under alkaline conditions, the silicon–oxygen bond will undergo a condensation reaction, and the macromolecular chain formed after the condensation of four-molecule DMDMS is mainly a linear chain structure (Figure 3c). The four-molecule MTMS has more hydroxyl groups that can participate in the condensation reaction, and after condensation, it can form a cyclic molecular chain structure (Figure 3d); therefore, it can be seen that the introduction of MTMS into the DMDMS reaction system can enrich the DMDMS polymer chains, and under appropriate reaction conditions, DMDMS and MTMS constantly undergo polycondensation reactions to form a three-dimensional network (Figure 4).

### 2.2. Hybrid Reaction of the Phenolic Silicone Aerogel

**Figure 5:**
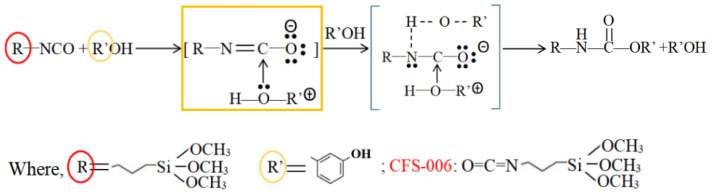
Equation of electrophilic addition between the isocyanate group and phenol hydroxyl group. The R and R’ in the red and orange circles represent different groups, and the orange box represents the process of nucleophilic addition reactions.

Isocyanate-based silane, due to its high activity and easy regulation, was selected as the crosslinking agent. The –N=C=O of isocyanate is a highly unsaturated group containing two continuous double bonds, and its chemical properties are very active. When the nucleophilic center of the resorcinol containing active hydrogen attacks the positive carbon atom, a nucleophilic addition reaction can occur, and the isocyanate group becomes a carbamate group. The specific reaction equation is shown in Figure 5.

**Figure 6:**
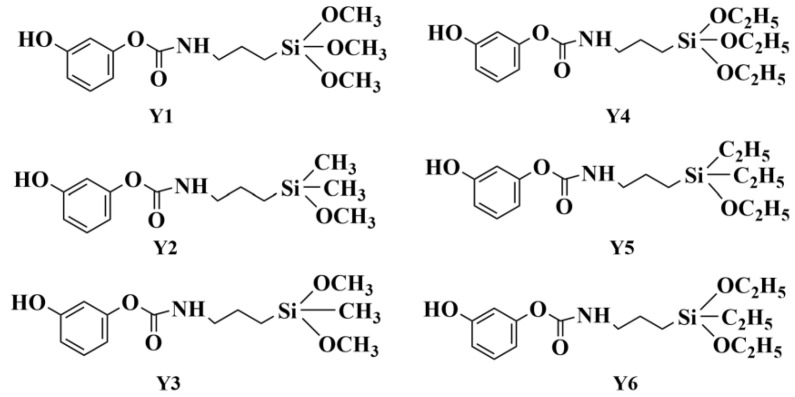
Isocyanate bridging molecular structures that have different functions.

**Figure 7:**
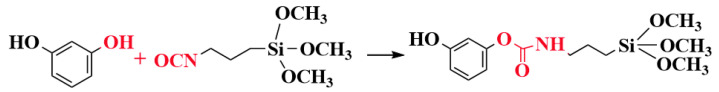
Synthesis equation for Y1.

The addition reaction between resorcinol and the isocyanate coupling agent can prepare a series of organic bridging molecules with different phenol hydroxyl and alkoxy functions. Figure 6 and Figure 7 show the structural formula of the bridging molecules that have different functions and the synthesis diagram for Y1, respectively.

**Figure 8:**
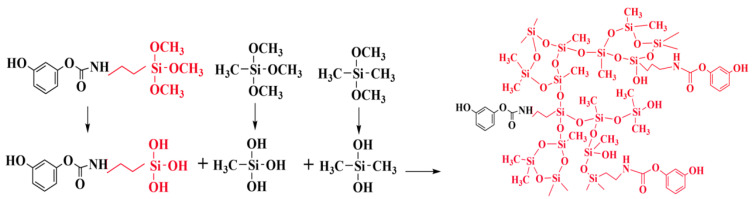
Hydrolysis polycondensation reaction of alkoxy end groups of the bridging molecules with silanes.

**Figure 9:**
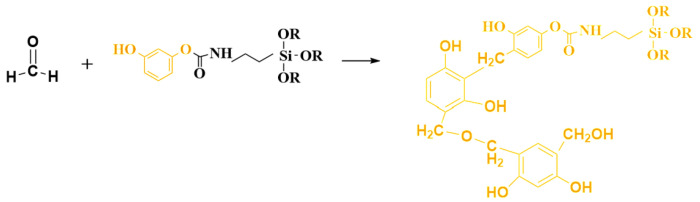
Electrophilic addition reaction of phenol hydroxyl end groups of the bridging molecules with formaldehyde.

**Figure 10:**
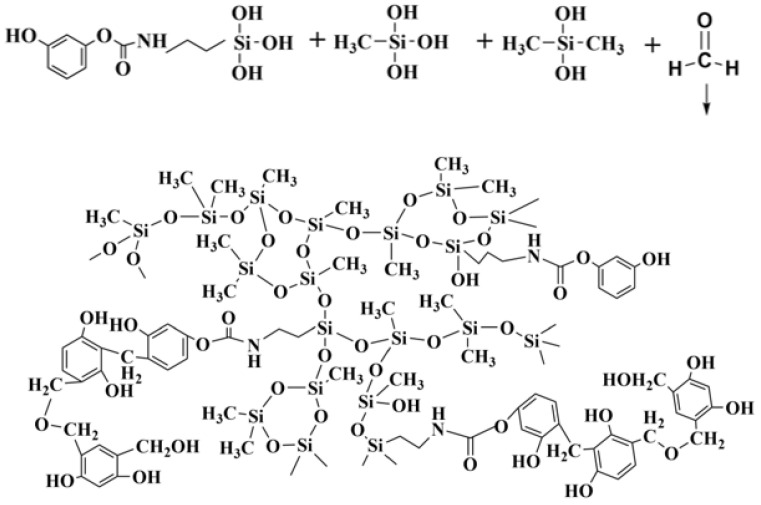
Reaction structure of the phenolic hybrid silicone aerogel.

The bridging molecule has good reactivity, and one end is silanoxy, which can hydrolyze and polycondensate with silane (Figure 8). The other end is the phenolic hydroxyl group, which has the activity of reacting with aldehyde, and the electrophilic addition reaction occurs at high temperature to generate phenolic polymer (Figure 9). Finally, the phenolic hybrid silicone aerogel system is generated, and the molecular formula is shown in Figure 10. In theory, the molecular structure generated by this chemical grafting method is stable and stronger than that of the traditional silicon and phenolic particles adsorbed by hydrogen bonds. The organic bridging molecule is evenly distributed in phenolic and polysiloxane solutions, and its molecular weight is larger. When the gel is further aged, it will inhibit the phase separation of the phenolic and polysiloxane components. The greater the amount added, the stronger the inhibition effect will be. It is feasible to control the morphology of the hybrid aerogel phase by changing the ratio of the phenolic monomer to the silicon source and the amount of coupling agent.

### 2.3. Surface Morphology

**Figure 11:**
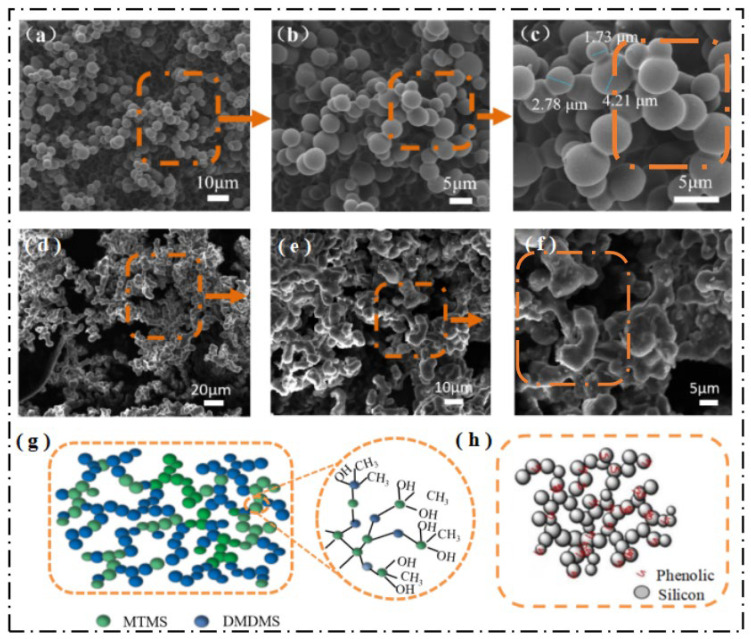
Microstructure of the DM and FAS aerogels: (**a**–**c**) SEM images of the DM aerogel; (**d**–**f**) SEM images of the FAS aerogel; (**g**) microscopic network structures of the DM aerogel; (**h**) microscopic network structures of the FAS aerogel. (**a**–**f**) show the SEM images of the DM and FAS aerogels at different magnifications, respectively, and the higher the magnification, the more explicit the observed particle structure.

Scanning electron microscopy (SEM) was used to observe the apparent morphology of the DM and FAS aerogels (Supplementary Materials), and the SEM images of the DM and FAS aerogels at different magnifications are represented in Figure 11a–c and Figure 11d–f, respectively, and show the pore and skeleton structures of the samples. It can be seen that both aerogels showed a honeycomb structure; the particle size of the aerogels was in the range of 1~5 μm, the particles were fused and connected with each other to form multiple “bead chains”, and the “bead chains” were intertwined with each other to form a three-dimensional network structure. The DM aerogel particle has a uniform shape and smooth surface, while FAS aerogel particle is interconnected by chemical cross-linking and has an irregularly shaped porous structure. Compared with the DM aerogel, the FAS aerogel’s inter-particle neck structure is more stable.

The microscopic network structures of the DM and FAS aerogels are represented in Figure 11g and Figure 11h, respectively. The molecular chain of the DM aerogel shown in Figure 11g consists of a large number of silica–oxygen covalent bonds and also contains a large number of methyl groups. The large silica particles of the FAS aerogel shown in Figure 11h have many fine organic phenolic particles attached to the surface, which is responsible for the formation of the irregular porous structure.

### 2.4. Mechanical Properties

**Figure 12:**
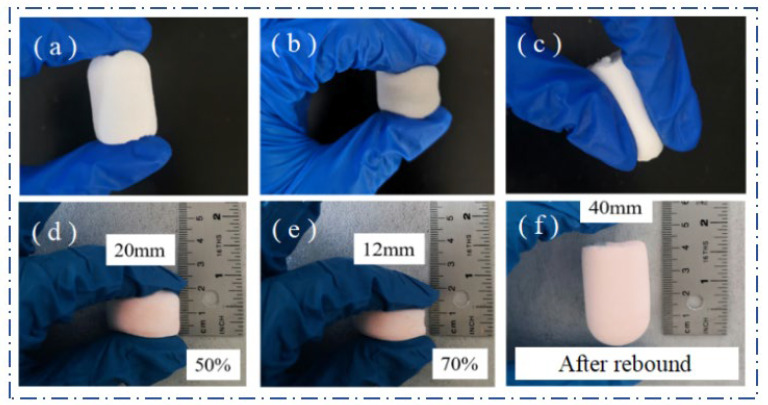
Compression performance of the aerogels: (**a**–**c**) the DM aerogel; (**d**–**f**) the FAS aerogel. (**a**–**f**) show the state of the DM aerogel and FAS aerogel after finger compression, respectively, and both have good compressibility.

**Figure 13:**
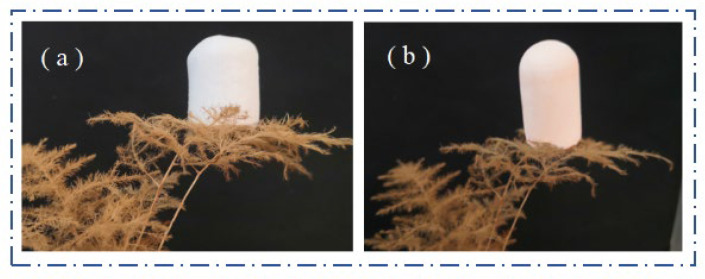
(**a**) The DM aerogel placed on leaves and (**b**) the FAS aerogel placed on leaves.

Pure SiO_2_ aerogels are usually in the form of irregular lumps or powder, and the produced DM aerogel and FAS aerogel are uniform monolithic lumps, as shown in Figure 12. Both the DM aerogel and FAS aerogel have the compressible property; Figure 12a–c demonstrates that the DM aerogel can be bent at will without any mechanical fracture after compression by the finger. Figure 12d–f shows that the FAS aerogel can still be restored to its original shape after finger compression to 70%; no fracture occurs during compression, there was no powder shedding, and it had good compression resilience. The density of the FAS aerogel is extremely low, only 0.118 g/cm^3^, and it contains a large amount of air inside. Figure 13 demonstrates the lightness of the DM aerogel and FAS aerogel, as when placed on a leaf, the leaf was not be deformed.

The DM aerogel and FAS aerogel have better flexibility than traditional silicone-based aerogels because the silicon source involved in the reaction is a multifunctional silane. The silicon atoms contain hydrolysable silicon–oxygen bonds, which inhibits the formation of a large number of silicon–oxygen bonds, reduces the cross-linking strength, and reduces irreversible shrinkage; a large number of the hydrophobic methyl groups do not participate in the hydrolysis and polycondensation, which not only improves the hydrophobicity of the material but also produces a “rebound effect” during drying to inhibit the shrinkage and collapse common to wet gels when drying [24,25].

### 2.5. Infrared Spectroscopic Analysis

**Figure 14:**
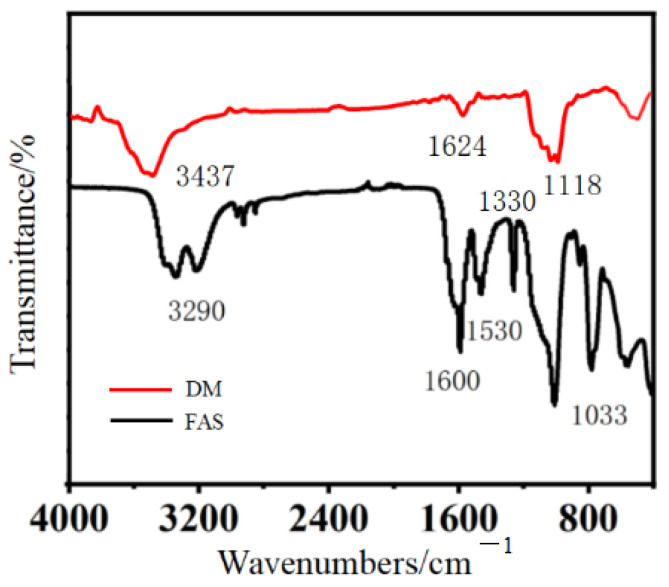
Infrared Spectrogram of the DM and FAS aerogels.

In order to explore the chemical structure of the DM and FAS aerogels, infrared tests were carried out on them, and the results are shown in Figure 14. The infrared spectrogram of the DM aerogel shows a strong characteristic peak near 1108 cm^−1^, which is a stretching vibration caused by the asymmetric stretching of Si–O–Si bonds in the silica-based aerogel network; the absorption peak near 3437 cm^−1^ can be attributed to the asymmetric –OH stretching vibration; and the micro-peak at 1624 cm^−1^ is the –OH deformation vibration of the adsorbed water molecules [26]. From the infrared spectrum of the FAS aerogel, it can be observed that the vibrational characteristic peak of the hydroxyl group on the aromatic ring appeared at 3290 cm^−1^ and the characteristic peak of –N=C=O was not observed at 2270 cm^−1^, which proved that the isocyanate group was involved in the chemical reaction to produce urethane –NH–C=OR. It can be observed from Figure 14 that 1330 cm^−1^, 1600 cm^−1^, and 1530 cm^−1^ are, respectively, the C=O stretching vibration peak, the N–H vibration peak, and the C–N absorption peak, and 1033 cm^−1^ is the characteristic peak of the silicon–oxygen bond.

### 2.6. Thermal Insulation Properties

The FAS aerogel has good thermal insulation at room temperature, with a thermal conductivity of 0.0318 W/(m·K), due to the complex skeleton that extends the path of heat conduction through the solid mechanism and the large number of pores that limit gas heat transfer.

**Figure 15:**
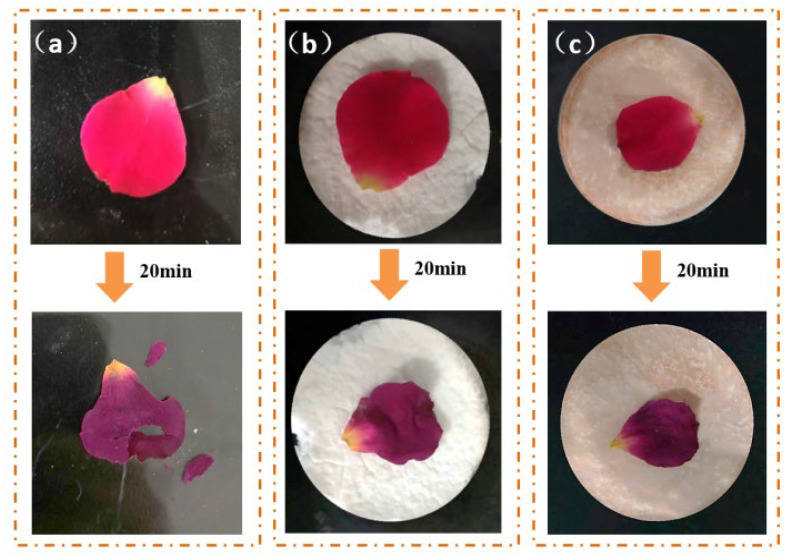
Thermal insulation of the aerogels at 150 °C for 20 min: (**a**) glass dish; (**b**) the DM aerogel; and (**c**) the FAS aerogel.

The DM and FAS aerogels were tested for heat insulation at high temperatures (Figure 15). After being treated at 150 °C for 20 min, petals placed on a glass dish would break when gently touched with tweezers. The weight loss rate of the petals was 97% and the surface temperature was 80.4 °C. The weight loss rate of the petals on the DM aerogel was 37% [27], and on the FAS aerogel was 36%. The petals showed obvious shrinkage, a low drying degree, and could maintain integrity with a light touch. The surface temperature was only 64.3 °C after 20 min, demonstrating good high temperature insulation.

### 2.7. Thermal Stability Analysis

**Figure 16:**
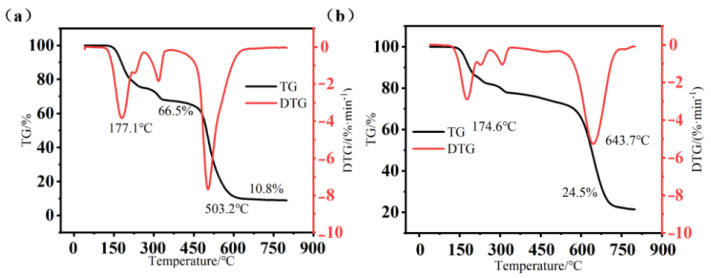
TG-DTG curves for (**a**) the DM aerogel and (**b**) the FAS aerogel.

Thermogravimetric analysis was used to explore the thermal stability of the DM and FAS aerogels. The test results are shown in Figure 16. Compared with the DM aerogel (Tmax 503.2 °C, residual rate 10.8%) [27], both Tmax and residual rate of the FAS aerogel are significantly increased, and the residual rate was 24.5% at 643.7 °C when Tmax was increased. It can be observed that the FAS aerogel has an obvious weight loss behavior between 150~400 °C, and derivative thermogravimetry (DTG) fluctuates greatly. This is because, although isocyanate stabilizes the structure of the hybrid aerogels, its thermal decomposition temperature is low, resulting in a large fluctuation in the thermal stability of the hybrid aerogels. Since the phenolic material introduced in the FAS aerogel has a stable structure and certain ablative resistance, the DTG curve becomes broad and generalized rather than sharp, which effectively inhibits the degradation of the silicone skeleton. It can be inferred that the introduction of the phenolic groups in the cross-linked network can effectively improve the thermal stability of the aerogel, and the material can be applied at 500 °C.

### 2.8. Hydrophobic Property Analysis

**Figure 17:**
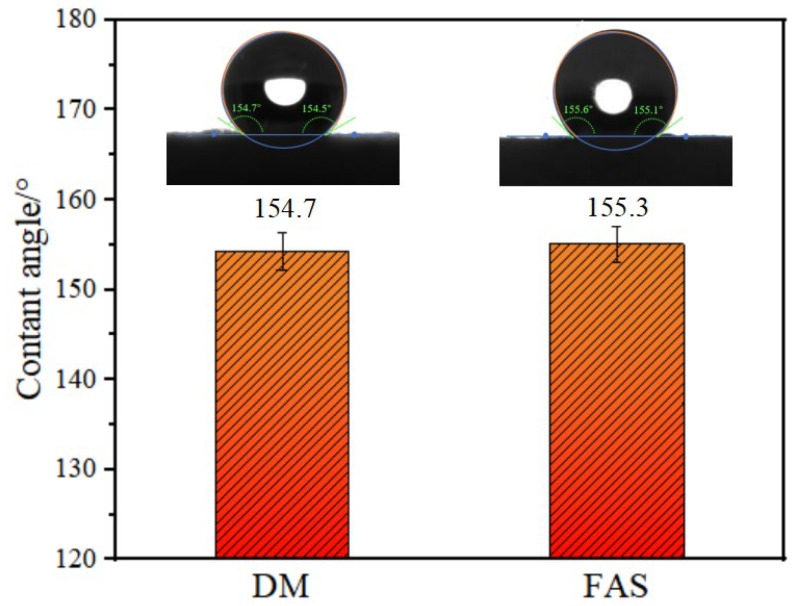
Contact angles of the DM and FAS aerogels.

Good hydrophobicity plays a positive role in maintaining thermal insulation of aerogel materials, especially aerogel insulation materials applied in humid environments [28]. The hydrophobic angle error bar graph is shown in Figure 17. The contact angles of the prepared DM and FAS aerogels reached 154.7°and 155.3°, respectively, which identify them as superhydrophobic materials. After the sol-gelation and drying processes, the methyl groups in DMDMS and MTMS were retained and covered the surface of the SiO_2_ skeleton, which gave the aerogel materials good superhydrophobic properties.

## 3. Materials and Methods

### 3.1. Materials

Anhydrous ethanol of analytical purity was purchased from Tianjin Windship Chemical Reagent Technology Co. (Tianjin, China); Glacial acetic acid of analytical purity was purchased from Tianjin Keres Fine Chemical Co. (Tianjin, China); Cetyltrimethylammonium chloride (CTAC), urea, MTMS, DMDMS, and CFS-006 of analytical purity were purchased from Tianjin Maidin Technology Co. (Tianjin, China); Formaldehyde of analytical purity (30%) and resorcinol of analytical purity were purchased from Tianjin Yifang Technology Co. (Tianjin, China).

### 3.2. Methods

#### 3.2.1. Preparation of the Phenolic Silicone Hybrid FAS Aerogel

**Figure 18:**
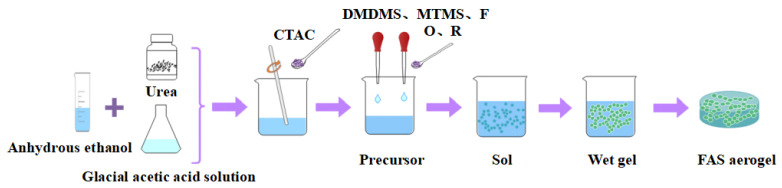
Preparation process for the FAS aerogel.

The mass ratio of fixed anhydrous ethanol, CTAC, urea, and glacial acetic acid (5 mM) was 1:1:5:14. The above materials were mixed and stirred for 10 min to obtain a uniform solution. The molar ratio of DMDMS and MTMS was 2:3, which was added to the mixed solution and stirred vigorously at room temperature for 45 min. At this stage, the solution is weakly acidic, and the silicon source is mainly hydrolyzed. An appropriate amount of isocyanate was added to the precursor solution, stirred for 30 min, and fully hydrolyzed under acidic conditions until the mixed solution was neutral to obtain the organosilicone sol. An appropriate amount of resorcinol and formaldehyde was added to the silica sol, and the phenolic silicone hybrid sol was obtained by stirring vigorously for 30 min. The isocyanate is O, resorcinol is R, formaldehyde is F, phenolic is RF, and the fixed molar ratios were nR:nF = 1:4, nRF:nSi = 1:430, and nO:nRF = 0.3:1. The preparation process is shown in Figure 18.

The obtained sol mixture was put into a closed container for aging at 90 °C. When the sol was tilted to 45° without any flow, the gelation was completed. After the wet gel was cooled to room temperature, it was washed in alcohol and dried under atmospheric pressure. The phenolic silicone hybrid aerogel was obtained after drying until the weight and volume did not change.

#### 3.2.2. Preparation of the Flexible Silicone DM Aerogel

**Figure 19:**
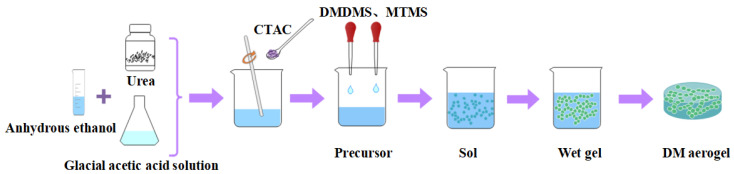
Preparation process for the DM aerogel.

The preparation method of the DMDMS and MTMS sol solution is the same as that of the FAS aerogel, without adding coupling agent CFS-006, resorcinol, and formaldehyde. After high temperature aging, washing in methanol and drying under atmospheric pressure, the DM aerogel was prepared. The preparation process is shown in Figure 19.

### 3.3. Characterization

#### 3.3.1. Shrinkage Test

The aerogel samples were all regular cylinders. The diameter and height of the wet gel before and after drying were measured with vernier calipers, and the change rate of the volume shrinkage was calculated.

#### 3.3.2. Microtopography Measurement

The microstructures of the aerogels were obtained using a Phenom XL SEM (Phenom-World Company, Eindhoven, The Netherland). Conductive tape was used to fix the sample to be measured on the SEM sample table, and then, the sample table was placed in the vacuum sample surface processor, and the sample was sprayed with gold for 50 s. Finally, the gold-sprayed samples were placed under the cold-field scanning electron microscope to observe the microstructure of the samples.

#### 3.3.3. Infrared Spectrum Test

The chemical structure of the sample was analyzed using a Frontier FT-IR (Pekin-Elmer Company, Waltham, MA, USA). The aerogel samples were dried for 20 min at 60 °C, ground into 40–60 mesh powder, and the samples was prepared by the KBr tablet method. The ratio of sample to KBr was 1:50, and the scanning range was 4000–400 cm^−1^.

#### 3.3.4. Thermogravimetric Test

The thermal effects and reactions of the samples from room temperature to 800 °C were measured with a STA449F3 thermogravimetric analyzer (Netzsch Company, Waldkraiburg, Germany) in a nitrogen atmosphere at a temperature rise rate of 10 °C/min.

#### 3.3.5. Hydrophobic Performance Test

The water contact angle (θ) was measured using a OCA15pro contact angle instrument from Dataphysics, Filderstadt, Germany. Deionized water was used to measure the contact angle of the solid surface. Each sample was measured in three different places in parallel. The contact angle reported was the average value. The sample length was 10 mm, the width was 10 mm, the height was 5 mm, and the volume of the water drop was 3 μL.

#### 3.3.6. Thermal Conductivity Test

The thermal conductivity was characterized by a TPS 2500S thermal constant analyzer (KegonasCompany, Shanghai, China) at 20 °C and a humidity 65%. The test polyimide (Kapton) film-covered probe model was 7531, the test module was a template module, the test time was 1 s, the heating power was 5 mW.

## 4. Conclusions

In order to improve the thermal stability of silicon-based aerogels, a phenolic-modified organosilicone hybrid aerogel was prepared by chemical grafting. CFS-006 was used as the cross-linking agent The end-group isocyanate group on one side was able to have an addition reaction with a phenol aldehyde, and the end-group alkoxy group on the other side could have a substitution reaction, which realized the combination of the organic phenol aldehyde and inorganic silica components through chemical bonding and solved the phase separation problem. When nRF:nSi = 1:430, nO:nRF = 0.3:1, and T = 90 °C, the density of the prepared FAS aerogel was only 0.118 g/cm^3^, with a uniform pink appearance and no phase separation problem. The FAS aerogel has good resilience (compressible up to 70%), low thermal conductivity (0.0318 W/(m·K), better high temperature insulation than the DM aerogel, and good hydrophobicity (155.3°). In addition, the introduction of an organic phenolic component improved the thermal stability, with the Tmax increasing to a 24.5% residual at 643.7 °C. In future work, the residual rate of hybrid aerogels should be further improved by increasing the content of the phenolic aldehyde. This work has positive significance for the future combination of aerogels and textiles in the preparation of firefighting protective clothing.

## Data Availability

The data that support the findings of this study are available from the corresponding author upon reasonable request.

## Supplementary Materials

The following supporting information can be downloaded at: https://www.mdpi.com/article/10.3390/molecules29204942/s1, Video S1: DM. Video S2: FAS.
